# A case of a cystic artery arising from the superior mesenteric artery with abnormal branching of the celiac trunk

**DOI:** 10.1186/s13104-017-2858-4

**Published:** 2017-10-30

**Authors:** Tomiko Yakura, Shogo Hayashi, Hayato Terayama, Takayoshi Miyaki, Takashi Nakano, Munekazu Naito

**Affiliations:** 1 Department of Anatomy, Aichi Medical University, 1-1 Yazakokarimata, Nagakute, Aichi 480-1195 Japan; 2 Department of Anatomy, Tokyo Medical University, Tokyo, Japan; 3 Department of Anatomy, Division of Basic Medicine, Tokai University School of Medicine, Kanagawa, Japan

**Keywords:** Calot’s triangle, Celiac trunk, Cystic artery, Inferior mesenteric artery, Superior mesenteric artery, Transverse colon

## Abstract

**Objective:**

The celiac trunk normally has three branches; i.e. the left gastric, splenic, and common hepatic artery. It is known that the right hepatic artery occasionally branches from the superior mesenteric artery, while the cystic artery arising from the superior mesenteric artery is extremely rare. A deeper understanding of cystic arterial variations is necessary for all physicians performing examinations and surgical procedures of the hepatobiliary system.

**Results:**

The cystic artery arising from the superior mesenteric artery was found in the cadaver of an 86-year-old woman during an anatomy dissection class at Aichi Medical University in 2015. In this case, the cystic artery ran along the dorsal side of the portal vein through Calot’s triangle to the gallbladder. The celiac trunk had four abnormal branches, one each to the left gastric, right hepatic, splenic, and left hepatic artery. The middle colic artery was absent and the left colic artery branching from the inferior mesenteric artery was distributed along the whole length of the transverse colon. In all cases of the cystic artery arising from the superior mesenteric artery, the vessel ran along the dorsal side of the portal vein; in addition, the right hepatic artery arose from the superior mesenteric artery.

## Introduction

The celiac trunk (CT) normally has three branches: the left gastric, splenic, and common hepatic arteries. The cystic artery (CyA) typically arises from the right hepatic artery, which branches from the proper hepatic artery divided from the common hepatic artery, and courses within Calot’s triangle to the gallbladder [1]. Understanding of the anatomical variations of the arterial supply to the gallbladder and liver is of great importance in all hepatobiliary surgical procedures. There have been reports of the CyA arising from the right hepatic artery, left hepatic artery, gastroduodenal artery, proper hepatic artery, common hepatic artery, and superior mesenteric artery (SMA) [2, 3]. Here, a rare case of the CyA, which arose from the SMA was encountered in an 86-year-old female cadaver during routine dissection class at Aichi Medical University in 2015. This case was associated with abnormal branching of the CT and inferior mesenteric artery (IMA).

## Main text

### Case presentation

**Fig. 1:**
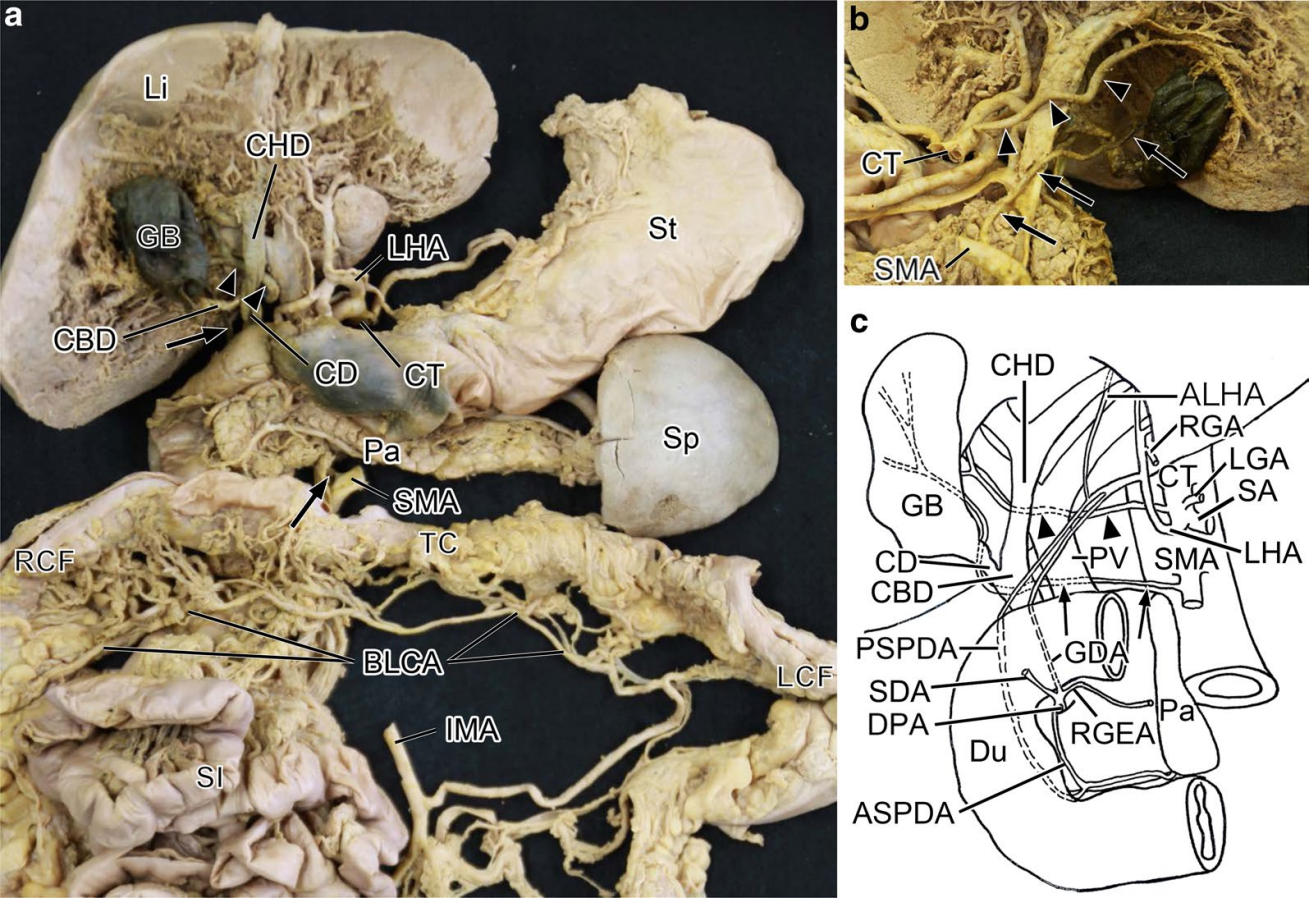
**a** Photograph showing the ventral aspect of the abdominal digestive organs, which were removed en bloc with their vessels from the abdominal cavity. **b** The dorsal view of the liver. **c** Schematic drawing showing the ventral aspect of the abdominal digestive organs. Arrows indicate the cystic artery. Arrow heads indicate the right hepatic artery

The cadaver was that of an 86-year-old Japanese female who died of a subcutaneous abscess. No macroscopic evidence of abdominal surgery was observed either on the body surface or in the abdominal or pelvic cavity. The abnormal branches from CT, SMA, and IMA were specifically observed during a student course on gross anatomical dissection at Aichi Medical University. The branching of the CT, SMA, and IMA was observed and recorded while paying close attention to the positional relationships of the CyA with these arteries. No intestinal malrotation was observed in the cadaver (Fig. 1a).

#### Four branches of CT

The CT had four branches: one each to the left gastric, right hepatic, splenic, and left hepatic artery (Fig. 1a, c). The left hepatic artery, which supplied the left hepatic lobe, originated 21 mm distal to the CT bifurcation and branched to the gastroduodenal and right gastric arteries and did not pass through Calot’s triangle (Fig. 1a, b). The right hepatic artery originated 15 mm distal to the CT bifurcation and ran along the dorsal side of the portal vein and common hepatic duct through Calot’s triangle and supplied the right hepatic lobe (Fig. 1a, b).

#### Cystic artery (CyA) and other branches of the superior mesenteric artery (SMA)

The CyA was branched from the SMA, which also branched the inferior pancreaticoduodenal, jejunal, ileal, ileocolic, and right colic arteries. The middle colic artery was not found in the branches of the SMA. The CyA originated 15 mm distal to the SMA bifurcation and branched 10 mm distal to the inferior pancreaticoduodenal artery. The CyA ran along the dorsal side of the portal vein through Calot’s triangle, and then distributed to the dorsal side of the gallbladder (Fig. 1b). The total length of the CyA was 130 mm. There was no obvious abnormality in vascular endothelial swelling, stenosis and thickness compared to normal cyst arteries.

#### Transverse colic distribution from the IMA

The IMA branched to the left colic artery, sigmoid arteries and superior rectal artery. The left colic artery originated 35 mm distal to the IMA bifurcation and passed through the left colic flexure until the vicinity of the right colic artery supplied from the descending colon to the whole transverse colon (Fig. 1a).

### Discussion

We encountered a rare case of the CyA arising from the SMA with abnormal branching of the CT and IMA in a Japanese female cadaver. The CyA typically arises from the right hepatic artery (79.02%) and courses within the Calot’s triangle to the right of the common hepatic duct [2]. Andall et al. [3] recently collated the experiences of 55 authors who examined 9800 cases and found only 20 instances in which the CyA arose directly from the SMA.

The CT in the present case had four branches. Adachi et al. [4] reported that the incidence of four branches was 0.8%. Moreover, in the present case, the left colic artery from the IMA developed to distribute the whole transverse colon, and the middle colic artery was absent. As this case is evidently rare, the frequency of incidence of such cases is unknown [5].

Embryologically, the primitive metameric intestinal arteries are connected by a longitudinal anterior anastomosis, and the longitudinal anastomosis vessels disappear between the CT and SMA as well as between the SMA and IMA during development [6]. The right hepatic artery usually originates from the fourth root (the future SMA) below the last of these three celiac branches [7]. The right hepatic artery arising from the CT exists in 2% cases [1]. In this case, it is supposed that the three arterial anomalies of the CT, SMA, and IMA were mutually related.

At present, laparoscopic cholecystectomy is widely accepted as the gold standard for the treatment of cholelithiasis. Blood vessel injuries during laparoscopic cholecystectomy, including CyA bleeding, result in conversion to open surgery in up to 1.9% of cases and mortality in about 0.02% [2]. Kaushik [8] reported that mortality goes up to nearly 15%, especially when the bleeding remains unrecognized. Rare variations cause an increase in the risk of unrecognized bleeding. Calot’s triangle, which is an important imaginary reference area in laparoscopic cholecystectomy, is crossed by the CyA. The CyA typically runs along the ventral side of the portal vein and the posterior side of the common bile duct. However, in all cases of the CyA arising from the SMA, including the present case, the CyA ran along the dorsal side of the portal vein. Moreover, in the present case, the CyA arose from the SMA and right hepatic artery and ran parallel to Calot’s triangle. Therefore, in such cases, it is difficult for physicians to judge the CyA arising from the SMA. It is known that the currently used computed tomography angiography is useful to detect such these abnormalities, non-invasively [3]. However, computed tomography angiography fails to provide an accurate and reliable depiction of cystic arterial vessels in 8% of cases. Safe laparoscopic cholecystectomy demands good knowledge of the anatomy of the CyA and its variations.

This is the 21st report of the CyA arising from the SMA. In all cases of the CyA arising from the SMA, including the present case, the CyA ran along the dorsal side of the portal vein. In laparoscopic cholecystectomy, an understanding of the normal anatomy of the biliary apparatus and common local anatomical variations is imperative.

### Limitations

There are many limitations of an anatomical variation in case report. Our greatest limitation was that we were unable to find out how much of the tissue is being supplied by each of these arteries as we do not have the infrastructure for such studies.

## Abbreviations

ALHA: accessory left hepatic artery
ASPDA: anterior superior pancreaticoduodenal artery
BLCA: branches of left colic artery
CT: celiac trunk
CBD: common bile duct
CD: cystic duct
CHD: common hepatic duct
DPA: dorsal pancreatic artery
Du: duodenum
GB: gall bladder
GDA: gastroduodenal artery
IMA: inferior mesenteric artery
LCF: left colic flexure
LGA: left gastric artery
LHA: left hepatic artery
Li: liver
Pa: pancreas
PSPDA: posterior superior pancreaticoduodenal artery
PV: portal vein
RCF: right colic flexure
RGA: right gastric artery
RGEA: right gastroepiploic artery
SA: splenic artery
SDA: supraduodenal artery
SI: small intestine
SMA: superior mesenteric artery
Sp: spleen
St: stomach
TC: transverse colon
